# Antinociceptive effects of hydroalcoholic extract from *Euterpe oleracea* Mart*.* (Açaí) in a rodent model of acute and neuropathic pain

**DOI:** 10.1186/s12906-015-0724-2

**Published:** 2015-07-02

**Authors:** Roberto T. Sudo, Miguel L. Neto, Carlos E.S. Monteiro, Rachel V. Amaral, Ângela C. Resende, Pergentino J.C. Souza, Gisele Zapata-Sudo, Roberto S. Moura

**Affiliations:** Program of Research in Drug Development, Institute of Biomedical Science, Federal University of Rio de Janeiro, UFRJ, Brazil. Av. Carlos Chagas Filho, 373 - Centro de Ciências da Saúde – bloco J, sala 14. Cidade Universitária – Ilha do Fundão, Rio de Janeiro, 21941-902 Brazil; Department of Pharmacology and Psychobiology, IBRAG, State University of Rio de Janeiro, UERJ, Av. 28 de setembro, 87 fundos 5° andar sala 7, Vila Isabel, Rio de Janeiro, 20551-030 Brazil; School of Pharmacy, Federal University of Para, Para, Brazil

**Keywords:** *Euterpe oleracea* Mart, Arecaceae, Hyperalgesia, Allodynia, Acute and chronic pain

## Abstract

**Background:**

Plants rich in flavonoids, such as açaí (*Euterpe oleraceae Mart*.), can induce antinociception in experimental animals. Here, we tested an extract obtained from the stones of açaí fruits (açaí stone extract, ASE), a native plant from the Amazon region of Brazil, in models of acute/inflammatory and chronic pain.

**Methods:**

**A**ntinociceptive effects of ASE were evaluated in the hot plate, formalin, acetic acid writhing, carrageenan, and neuropathic pain models, as well as in thermal hyperalgesia and mechanical allodynia models induced by spinal nerve ligation. Antinociceptive activities were modulated by the administration of cholinergic, adrenergic, opioid, and L-arginine-NO antagonists**.**

**Results:**

Oral administration of ASE (30, 100, or 300 mg.kg^−1^) dose-dependently reduced nociceptive responses to acute/inflammatory pain in mice, including thermal hyperalgesia, acetic acid-induced writhing, and carrageenan-induced thermal hyperalgesia. Moreover, ASE reduced the neurogenic and inflammatory phases after intraplantar injection of formalin in mice. The antinociceptive effect of ASE (100 mg · kg^−1^) in a hot plate protocol, was inhibited by pre-treatment with naloxone (1 mg · kg^−1^), atropine (2 mg · kg^−1^), yohimbine (5 mg · kg^−1^), or L-NAME (30 mg · kg^−1^). Furthermore, ASE prevented chronic pain in a rat spinal nerve ligation model, including thermal hyperalgesia and mechanical allodynia.

**Conclusion:**

ASE showed significant antinociceptive effect via a multifactorial mechanism of action, indicating that the extract may be useful in the development of new analgesic drugs.

## Background

Pain can reduce normal activities and negatively impact quality of life. Current options for the pharmacological treatment of pain include non-steroidal anti-inflammatory drugs and opioids, which unfortunately cause several side effects. The biodiversity present in countries like Brazil represents a potentially important source for development of new analgesic compounds [1].

*Euterpe oleracea* Mart., popularly known as “açaí”, belongs to the family *Arecaceae* and is widely distributed in the Amazon region of Brazil. The fruit is an important source of food and is used as a medicinal plant for fever, pain, inflammation and anemia treatment [2].

Açaí fruits are rich in anthocyanic compounds (cyanidin 3-O-rutinoside) and other polyphenols, such as epicatechine, catechine homoorientin, orientin, isovitexin, and taxifolin deoxyhexose [3, 4], which have important biological effects.

Previously, we demonstrated that the hydro-alcoholic extracted from açaí stones (açaí stone extract, ASE), which is rich in polymeric proanthocyanidins, have important vasodilatory [5], antihypertensive [6], antioxidant [7], and anti-inflammatory [8] activities. As extracts from plants rich in flavonoids can show antinociceptive effects [9, 10], we tested the effects of ASE in acute and chronic models of pain, as well as the mechanisms underlying these effects.

## Methods

### Preparation of ASE

*E. oleracea* Mart*.* fruits (açaí) were obtained from Amazon Bay (Belém do Pará, Brazil; excicata number 29052, Museu Goeldi-Belem do Pará). Hydro-alcoholic extracts were obtained from a decoction of the seeds of the fruits as previously described by Moura et al. [8]. Briefly, 200 g of açaí stone were boiled in 400 ml of distilled water for 5 min, mixed for 2 min, and then boiled again for 5 min. The decoction was cooled to room temperature and extracted by addition of 400 ml of ethanol with shaking for 2 h. The extract was stored in dark bottles inside a refrigerator (4 °C) for 10 days. After this maceration period, hydroalcoholic extracts of açaí were filtered through Whatman filter paper. Ethanol was evaporated by using a rotary evaporator (Fisatom Equipamentos Científicos Ltda São Paulo, São Paulo, Brazil) under low pressure at 55 °C. The extract was lyophilized (LIOTOP model 202, Fisatom Equipamentos Científicos Ltda São Paulo) at temperatures from −30 to −40 °C and under a vacuum of 200 mmHg, and frozen at −20 °C, until use. Typically, 100 g of stone yielded approximately 5 g of lyophilized extract.

ASE was analyzed on an RP-18 column (250 mm × 4 mm, 5 μm particles) according to a procedure reported by Peng et al. [11]. Elution was conducted with solvents A (0.2 % v/v phosphoric acid) and B (82 % v/v acetonitrile, 0.04 % v/v phosphoric acid) at a flow rate of 1 ml.min^−1^. Ultraviolet–visible (UV–vis)-DAD absorption spectra were recorded on-line during High-Performance Liquid Chromatography (HPLC) analysis. The HPLC elution profile of ASE can indicate the presence of proanthocyanidins [11]. The peak eluting at 37.2 min corresponded to catechin, as confirmed by co-injection of a standard and by comparison of the UV absorption spectra. The late elution (at 54.7 min) and UV spectrum of the main peak are consistent with the presence of polymeric proanthocyanidins, as previously described [8].

### Animals and housing conditions

Male Swiss mice (18–25 g) and male Wistar rats (180–220 g), obtained from Vital Brasil Institute and the Federal University of Rio de Janeiro, respectively, were housed under a 12 h light–dark cycle at 21 °C and 60 % humidity, with food and water ad libitum. Protocols were reviewed and approved by the institutional Animal Care and Use Committee (CEUA, Ref. #DFBCICB061).

### Drugs

Atropine, carrageenan, acetylsalicylic acid, indomethacin, formaldehyde and L-nitro arginine methyl ester (L-NAME) were purchased from Sigma (St Louis, MO, USA). Yohimbine hydrochloride was purchased from Tocris (Ellisville, MO, USA). Tramadol, naloxone, amitriptyline hydrochloride and morphine sulfate were donated by Cristália Produtos Químicos e Farmacêuticos Ltda (Itapira, SP, Brazil). ASE was dissolved in distilled water (10 mg.ml^−1^, stock solution).

### Hot plate test

**Fig. 1:**
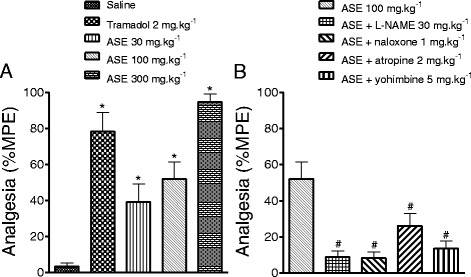
**a** Effect of ASE (30, 100 and 300 mg.kg^−1^, p.o.) and tramadol (2 mg.kg^−1^, p.o) in the hot plate test, **b** Evaluation of mechanism of action of ASE. The bars represent the mean ± SEM (*n* = 10). *P < 0.05 versus saline, ^#^ *P* < 0.05 versus ASE group. ANOVA followed by Newman-Keuls test

The hot plate test in mice [12] was used to test the effect of orally administered ASE (30, 100 or 300 mg.kg^−1^) on pain responses mediated by the central nervous system (CNS). Oral tramadol (2 mg.kg^−1^) was used as a positive control. Withdrawal latency (reaction time of the animal when placed on a surface heated to 52 °C) was measured before and 30 min after oral administration of either saline tramadol (2 mg.kg^−1^) or ASE (30, 100 or 300 mg.kg^−1^). Additional measurements were performed every 15 min up to 120 min to determine the maximum possible effect (%MPE), which occurred 20–25 min after ASE administration. Analgesic activity was calculated as the %MPE by using the formula: %MPE = [(latency observed) – (latency control) x 100] / [(cut-off) – (latency control)] (Fig. 1).

To investigate the possible mechanisms involved in ASE activity animals received pre-administration of the following antagonists: 30 mg.kg^−1^ N^ω^-nitro-L-arginine methyl ester (L-NAME, selective NOS inhibitor), 1 mg.kg^−1^ naloxone (opioid antagonist), 2 mg.kg^−1^ atropine (muscarinic antagonist), or 5 mg.kg^−1^ yohimbine (α_−2_ adrenoceptor antagonist). A 100 mg.kg^−1^ dose of ASE, produced a MPE of 50 % and was used for the mechanism of action experiments.

### Formalin-induced hind paw-licking

The antinociceptive effect of ASE on neurogenic and inflammatory pain was tested by using the formalin test in mice [13]. Formalin (2.5 %, 20 μl) was administered by intraplantar injection into the right hind paw 15 min after oral administration of saline, acetylsalicylic acid (150 mg. kg^−1^), morphine sulfate (30 mg.kg^−1^) or ASE (30, 100, or 300 mg.kg^−1^). The duration of licking and biting of the injected paw was monitored over 0–5 min (early phase, neurogenic pain response) and 15–30 min (late phase, inflammatory pain response).

### Carrageenan-induced pain

Carrageenan-induced thermal hyperalgesia was evaluated in mice as described [12, 14]. Animals were placed in transparent boxes on a glass surface, and a radiant heat stimulus was applied through the glass onto the hind paws until withdrawal. Latency was defined as the time between heat application and hind paw withdrawal. Peripheral inflammation was induced by intraplantar injection of carrageenan (1 %, 20 μl) into the right hind paw at time zero. The latency of each animal to react to the thermal stimulus was measured at different time points before (control measure) and after carrageenan injection. Saline, acetylsalicylic acid (150 mg.kg^−1^), or ASE (30, 100, or 300 mg.kg^−1^) was administered orally 15 min before carrageenan. A cut-off time of 15 s was used to avoid tissue damage.

### Acetic acid-induced writhing

Mice received intraperitoneally (i.p.) administered acetic acid (0.6 %, 10 μl.g^−1^ v/v), as previously reported [15], and were placed in a box (40 x 30 x 25 cm) in a quiet, illuminated room. The resulting abdominal contortions (writhes) were counted for 20 min, beginning 10 min after acetic acid administration, as previously described [16]. Saline, the reference drug indomethacin (2 mg.kg^−1^) or ASE (30, 100, or 300 mg.kg^−1^) was administered orally 15 min before acetic acid.

### Spinal nerve ligation (SNL)

Neuropathic pain signs (thermal hyperalgesia and mechanical allodynia) were induced by SNL as described [17]. Briefly, after anesthesia with ketamine (100 mg.kg^−1^ i.p.) and xylazine (5 mg.kg^−1^ i.p.), Wistar rats (180–220 g), were placed in the prone position. The right L5 spinal nerve was isolated and tightly ligated with 6.0 silk threads. After the procedure, the wound was sutured. Animals were individually housed after surgery for the remainder of the study.

### Antinociceptive effect on SNL-induced thermal hyperalgesia and mechanical allodynia

Thermal hyperalgesia was assessed using latency of paw withdraw [14, 18] from a radiant heat source applied to the plantar surface of the hind paws. Animals were placed in transparent acrylic boxes for 20–30 min to acclimatize before application of radiant heat through the glass flooring. Latency from stimulus onset to paw withdrawal was measured across three trials with a cutoff of 30 s.

Mechanical allodynia was assessed by using a digital version of the Von Frey filaments [18]. Rats were placed in individual acrylic boxes for 30 min to acclimatize. Stimuli were applied to the plantar region of the hind paw, and the withdrawal threshold was assessed across five trials with a cutoff of 120 g. Control measurements were taken before and 7 days after SNL. Rats were subjected to thermal hyperalgesia and mechanical allodynia tests to confirm the success of SNL surgery and the onset of neuropathic pain. After daily treatment with ASE for 7 days, pain tests were repeated.

### Statistical analysis

Data are reported as the mean ± standard error of the mean (S.E.M.). One-way ANOVA followed by the Newman-Keuls test was used to analyze the effects of ASE on the hot plate test, formalin-induced pain, and acetic acid-induced writhing. Two-way ANOVA followed by the Bonferroni post-hoc test was used to analyze the effects of ASE on the carragenin-induced pain and on the SNL experiments. Data were graphed and statistically analyzed by using GraphPad Prism 5.0. Differences were considered significant when the *p* value <0.05.

## Results and discussion

### Hot plate test

Treatment with 30, 100, or 300 mg.kg-1 ASE dose-dependently increased the %MPE to 39.1 ± 10.0, 51.9 ± 9.5, or 94.7 ± 4.4 %, respectively (Fig. 1a, n = 10 per group, *p* < 0.05). The %MPE was also increased by tramadol (2 mg.kg^−1^) to 78.3 ± 10.3 % (n = 10, *p* < 0.05). Pre-treatment with i.p. administration of L-NAME (30 mg.kg^−1^), naloxone (1 mg.kg^−1^), yohimbine (5 mg.kg^−1^) or atropine (2 mg.kg^−1^) reduced the antinociceptive effect of ASE (100 mg.kg^−1^) from 51.9 ± 9.5 to 8.9 ± 3.3, 8.3 ± 3.3, 13.6 ± 4.1, or 26.2 ± 6.8 %, respectively (Fig. 1b, n = 10 per group, *p* < 0.05).

Nociception induced by thermal stimulation (hot plate test) is used to evaluate antinociceptive agents that act centrally but not peripherally [19]. This test involves various physiological systems, including cholinergic, adrenergic, opioid, and L-arginine/NO, which may be targets for antinociceptive compounds.

The importance of the sympathetic nervous system in pain modulation has been known since 1904, when Weber [20] demonstrated the antinociceptive effect of epinephrine injected in the spinal cord of a cat. Intrathecal or intraperitoneal administration of α2-adrenoceptor agonists induces significant antinociceptive effects in the hot plate test in rodents [21]. Here, the α2-adrenoceptor antagonist yohimbine inhibited the antinociceptive effect of ASE, supporting involvement of the adrenergic system on pain modulation, consistent with others flavonoids [22]. The antinociceptive effect of ASE is probably dependent on flavonoids content, because flavones [23], and quercetine [24] have similar effect in animals. Specifically, polymeric proanthocyanidins, which are common compounds in our extract, may underlie the antinociceptive effects, as seem with proanthocyanidins obtained from *Croton celtidifolius* bark [9].

Morphine is considered to be the gold standard drug for systemic pain treatment. However, prolonged use of morphine induces tolerance and hyperalgesia. In the present study naloxone, an opioid antagonist blocked the anti-nociceptive effects of ASE. Opioid mechanisms also modulate the antinociceptive effects of flavones compounds [23] and quercetin [22]. Muscarinic cholinergic receptors are present along the pain pathway from the dorsal root ganglia to somatosensory cortex [25], and muscarinic agonists have antinociceptive effects in rodents [18]. Inhibition of muscarinic receptors by atropine reduced, but did not abolish the antinociceptive effect of ASE. This finding suggests that cholinergic mechanisms may mediate these activities.

The L-arginine–nitric oxide (NO)/cyclic guanosine monophosphate (cGMP) pathway also modulate pain responses [26]. NO activates soluble guanylyl cyclase, leading to the production of cGMPn which activates cGMP-dependent protein kinase to open ATP-sensitive K^+^ channels, leading to neuronal hyperpolarization and spinal and peripheral antinociception [27]. In this study, the NO synthesis inhibitor L-NAME inhibited the antinociceptive effect of ASE. This inhibition demonstrates the involvement of the L-arginine-NO-pathway to the antinociceptive activities of ASE. Inhibition of NO synthesis antagonizes the activities of several antinociceptive compounds [28].

Taken together, these results indicate that ASE has an antinociceptive effect that is modulated by the cholinergic, adrenergic, opioid, and L-arginine-NO pathways. In addition, reactive oxygen species can enhance nociceptive responses [29], and ASE may block these responses via antioxidant activities and increasing NO-synthase to release NO [30].

### Formalin-induced hind paw-licking test

**Fig. 2:**
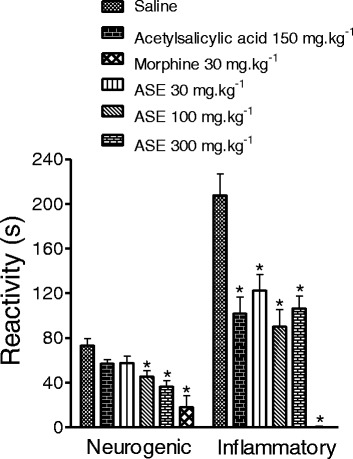
Effect of ASE and acetylsalicylic acid on the formalin test. The bars represent mean ± SEM (*n* = 10). *P < 0.05 versus saline. ANOVA followed by Newman-Keuls test

The total amounts of time spent licking, scratching, or biting during the neurogenic and inflammatory phases after intraplantar injection of formalin were 73.1 ± 6.1 s and 207.8 ± 19.0 s, respectively (Fig. 2). Reactivity in the neurogenic phase was not affected by oral administration of the lowest doses of ASE (30 mg.kg^−1^) or acetylsalicylic acid (150 mg.kg^−1^), but was reduced by higher doses (100 and 300 mg.kg^−1^ ASE) to 45.6 ± 5.0 s and 36.4 ± 5.3 s, respectively (*p* < 0.05). Reactivity in the inflammatory phase was reduced by acetylsalicylic acid to 101.9 ± 14.9 s and by (30, 100, or 300 mg.kg^−1^ ASE) to 122.5 ± 14.5 s, 90.1 ± 15.2 s and 106.4 ± 11.0 s, respectively ( *p* < 0.05).

Intraplantar injection of formalin in rodents induces nociceptive-related behavior when assessed over two temporally distinct phases [13]. The first phase is induced by a direct activation of peripheral afferent C-fibers. The second phase is mediated by ongoing stimulation of nociceptors by inflammatory mediators (serotonin, histamine, bradykinin, NO, and prostaglandins) released from injured tissue, leading to activity-dependent sensitization of CNS neurons within the dorsal horn [31]. Local anesthetics and morphine inhibit the first phase whereas NSAIDs inhibit the second inflammatory phase. In this study, we found that ASE inhibited the first phase, probably due to interaction with CNS targets. ASE reduced reactivity in the second phase; this finding suggests that ASE has anti-inflammatory activities, perhaps via inhibition of cyclooxygenase 1 and 2 [4].

### Carrageenan-induced pain test

**Fig. 3:**
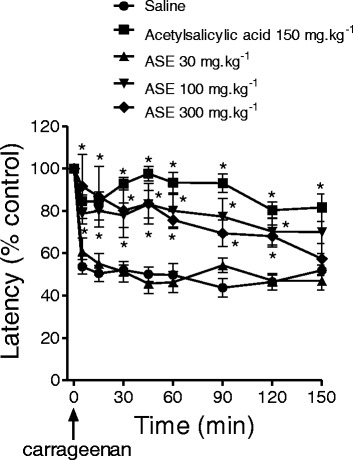
Effect anti-hyperalgesic of ASE and acetylsalicylic acid on the carrageenan test. The points represent the mean ± SEM (*n* = 10). *P < 0.05 versus saline. Two-way ANOVA followed by Bonferroni post hoc test

Intraplantar administration of carrageenan reduced paw withdrawal latency to heat stimulation to 55.4 ± 5.7 % of control (Fig. 3). The effect of carrageenan was noted 5 min after administration, sustained for 150 min, and not affected by oral administration of ASE (30 mg.kg^−1^). However, higher doses of ASE (100 and 300 mg.kg^−1^) or acetylsalicylic acid (150 mg.kg^−1^) reduced the effect of carrageenan on paw withdrawal latency.

After carrageenan-induced inflammation, noxious stimuli elicit an enhanced pain response (hyperalgesia) [14]. This enhanced synaptic transmission is essential for central sensitization. ASE prevented the appearance of this sensitization, supporting its antinociceptive effects in inflammatory pain. Some flavonoids in açaí are modulate proinflammatory cytokine production [32]. Carrageenan stimulates the release of tissue necrosis factor (TNF)-α, interleukin (IL)-1β and IL-6, with subsequent increases in COX products and IL-8, to stimulate local production of sympathetic amines [33]. Therefore, ASE may block the cascade of cytokine release induced by carrageenan-induced sensitization to produce analgesia in inflammatory pain.

### Acetic acid-induced writhing test

**Fig. 4:**
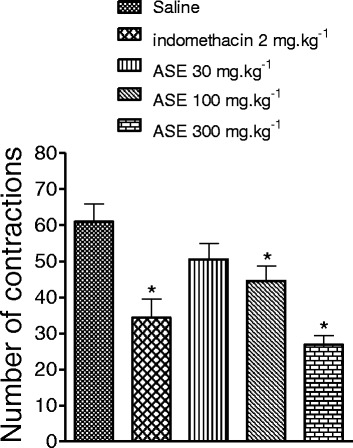
Effect of ASE and indomethacin on the acetic acid-induced writhing test. The number of writhing was evaluated during 30 minutes. The bars represent the mean ± SEM (*n* = 10). *P < 0.05 versus saline. ANOVA followed by Newman-Keuls test

ASE at 100 and 300 mg.kg^−1^ dose-dependently reduced the number of abdominal contractions in response to acetic acid from 61.0 ± 4.8 (saline) to 44.5 ± 4.2 and 26.9 ± 2.5, respectively (*p* < 0.05). This effect was not significant at the lowest dose of ASE (30 mg.kg^−1^), which slightly reduced contractions to 50.5 ± 4.4. The reference drug indomethacin (2 mg.kg^−1^) reduced contractions to 34.4 ± 5.1 (Fig. 4).

The acetic acid-induced writhing test is a screening tool for assessment of antinociceptive and anti-inflammatory agents [34]. Intraperitoneal injection of acetic acid increases pain mediators, such as prostaglandins, lipoxygenase, cyclooxygenase, histamine, serotonin, bradykinin, substance P, IL-1β, IL-8, and TNF-α [34, 35], which increase vascular permeability and reduce the nociceptive threshold, causing stimulation of nociceptive terminals to induce abdominal writhing. The writhing response starts a few minutes after acetic acid injection. Reduction of this behavior is used to test the efficacy of drugs with visceral antinociceptive activity [36]. We measured the writhing response for 20 min starting 10 min after acetic acid injection to avoid counting stress reaction of the animal due to manipulation. We found similar writhing levels to other studies that measured the reaction for 30 min starting 5 min after acetic acid administration [16, 37]. Pre-treatment with ASE reduced the acetic acid-induced writhing response, suggesting reduced synthesis or release of pain modulators.

### SNL-induced thermal hyperalgesia and mechanical allodynia

**Fig. 5:**
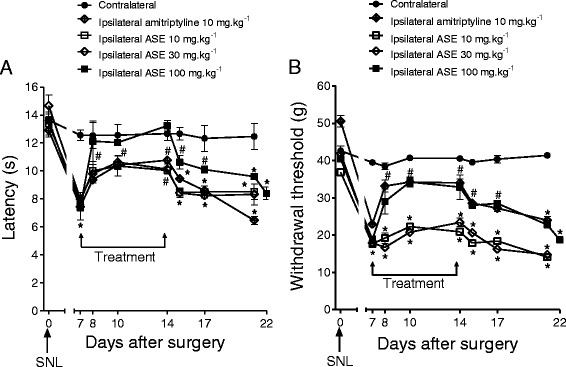
Antinociceptive effect of ASE or amitriptyline in SNL rats. The ASE and amitriptyline were once daily administered by gavage during 7 days. **a**) Latency in response to thermal stimulation and **b**) Withdrawal threshold in response to mechanical stimulation applied to the paw of rats submitted to SNL. The points represent the mean ± SEM (n = 4). *P < 0.05 versus day 0; ^#^ *P* < 0.05 versus day 7. Two-way ANOVA followed by Bonferroni post hoc test

ASE (10, 30, or 100 mg.kg^−1^) dose-dependently prevented development of thermal hyperalgesia and mechanical allodynia in SNL rats on the ipsilateral side (Fig 5a and b), but no effect was observed on the contralateral side. At 7 days after surgery, the thermal withdrawal duration was reduced from 13.6 ± 0.5 s to 7.4 ± 0.9 s (n = 4). ASE had significant effects from day 1 to 7 of treatment, reaching 13.2 ± 0.4 s. Treatment with 10 or 30 mg.kg^−1^ ASE was as effective as 10 mg.kg^−1^ amitriptyline. The mechanical withdrawal threshold was reduced 7 days after surgery from 40.5 ± 0.6 g to 18.8 ± 1.0 g. After 7 days of treatment, ASE (100 mg.kg^−1^, n = 4) increased this threshold to 32.9 ± 3.2 g, similar to amitriptyline (10 mg.kg^−1^, n = 4). ASE had no effect on withdrawal duration or withdrawal threshold in the contralateral paw (Fig. 5).

Chronic pain with neuropathic features affects 7-8 % of the general population [38]. Unfortunately, current pharmacotherapies used to treat the main symptoms of this disorder, hyperalgesia and allodynia, are not completely effective. Oral administration of ASE over 7 days prevented the development of thermal hyperalgesia and mechanical allodynia in rats with SNL. Analgesic effects of ASE in this model were observed from 1 to 7 days after treatment with no signs of tolerance, which is a drawback of morphine [39]. Furthermore, side effects such as sedation were not observed after prolonged ASE treatment, providing an advantage over amitriptyline, which is sedative in humans [40]. A combination of the CNS and anti-inflammatory effects of ASE may underlie the antinociceptive effects in rats subjected to SNL.

Flavonoids such as the polyphenolic compounds rutin and quercetin have anti-inflammatory [41], analgesic [42], and antioxidant [43] effects. SNL is a neuropathic pain model used in rats that mimics the pain sensations experienced by human patients [44]. ASE had comparable efficacy to the clinical drug amitriptyline, in treating SNL-induced neuropathic pain. Others flavonoids can impact animal models of neuropathic pain. For example, Azevedo et al. [24] showed that rutin and quercetin prevented thermal and mechanical nociceptive responses in oxaliplatin-induced neuropathic pain in mice by mediating oxidative stress-induced damage.

## Conclusions

The present study demonstrates a significant and potent antinociceptive effect of oral ASE. The mechanism of this antinociceptive effect is not completely understood, but probably involves various pathophysiological systems. These findings indicate the possibility for development of a new analgesic drug.
